# Intracellular transport, molecular motors, KIFs and related diseases

**DOI:** 10.1186/1471-2164-15-S2-O19

**Published:** 2014-04-02

**Authors:** Nobutaka Hirokawa

**Affiliations:** 1 Department of Cell Biology and Anatomy, Graduate School of Medicine, University of Tokyo; 2 Center of Excellence in Genomic Medicine Research, P.O. Box: 80216 Jeddah 21589, King Abdulaziz University, Kingdom of Saudi Arabia

## 

The intracellular transport is fundamental for cellular functions, morphogenesis and survival in general including neurons composed of a very long axon and dendrites.

We discovered most of the kinesin superfamily motor proteins, KIFs, 45 genes in mammals, elucidated their molecular structures and functional roles by molecular cell biology, molecular genetics, biophysics and structural biology and successfully disclosed the mechanism of intracellular transport fundamental for neuronal functions.

In the axon and dendrites KIFs transport their cargos such as synaptic vesicle precursors (KIF1A/KIF1Bbeta), mitochondria (KIF1Balpha/KIF5s), plasma membrane proteins (KIF3/KIF5s), NMDA glutamate receptors (KIF17), AMPA receptors (KIF5s) and mRNA with large protein complex (KIF5s). KIFs mainly recognize and bind their cargoes through adaptor protein complex and release them via phosphorylation of KIFs or GTP hydrolyses of cargo G-protein. Furthermore, using molecular genetics we successfully uncovered that KIFs play significant roles for fundamental physiological phenomena in development and functions of nervous system and that deletion of KIFs causes certain diseases by clarifying followings: 1) KIF1A/KIF1B beta hetero mice serve as a model for neuropathy, 2) KIF3 determines left/right asymmetry by generating cilia and nodal flow in the node of early embryos, 3) KIF17 plays a fundamental role on learning and memory by not only transporting NMDA glutamate receptor in dendrites but also controlling transcription and translation of KIF17 and NMDA receptor mRNAs by enhancing phosphorylated CREB, 4) KIF1A is essential for hippocampal synaptogenesis and learning enhancement in an enriched environment, 5) KIF2A is fundamental for brain wiring by controlling unnecessary elongation of growth cones by depolymerizing microtubules, 6) KIF4 plays a crucial role in the activity-dependent survival of postmitotic neurons in brain development by regulating poly(ADP-ribose) polymerase-1 activity, 7) KIF26A is essential for enteric neuronal development by regulating GDNF-Ret signaling, 8) KIF3 suppresses tumorigenesis by transporting beta-catenin from Golgi to plasma membrane for serving as cell-cell adhesion molecules, inhibiting its accumulation in the nucleus and suppressing hyper proliferation of progenitor cells, 9)KIF5A is essential for GABAa receptor transport and KIF5A deletion causes epilepsy, 10)KIF19A is a microtubule depolymerizing KIF for ciliary length control and its deletion causes female infertility and hydrocephalus based on affected fluid flows, 11)KIF13A transports serotonin receptors to plasma membranes and its deletion causes elevated–anxiety phenotypes.

Thus, KIFs play significant roles not only at cellular level, but also in brain function and development. Further, their malfunctions cause diseases such as neuropathy, epilepsy, dementia, elevated anxiety, tumor, megacolon and hydrocephalus.

